# Evolving Technologies in Gastrointestinal Microbiome Era and Their Potential Clinical Applications

**DOI:** 10.3390/jcm9082565

**Published:** 2020-08-07

**Authors:** Abraham Ajayi, Tolulope Jolaiya, Stella Smith

**Affiliations:** 1 Department of Molecular Biology and Biotechnology, Nigerian Institute of Medical Research, Yaba, Lagos 101212, Nigeria; ajayiabraham2013@gmail.com; 2 Department of Microbiology, University of Lagos, Akoka 101212, Nigeria; oshuntee@yahoo.com; 3 Department of Biological Sciences, Mountain Top University, Ogun 110106, Nigeria

**Keywords:** gastrointestinal, microbiota, technology, therapy, high-throughput

## Abstract

The human gastrointestinal microbiota (GIM) is a complex and diverse ecosystem that consists of community of fungi, viruses, protists and majorly bacteria. The association of several human illnesses, such as inflammatory bowel disease, allergy, metabolic syndrome and cancers, have been linked directly or indirectly to compromise in the integrity of the GIM, for which some medical interventions have been proposed or attempted. This review highlights and gives update on various technologies, including microfluidics, high-through-put sequencing, metabolomics, metatranscriptomics and culture in GIM research and their applications in gastrointestinal microbiota therapy, with a view to raise interest in the evaluation, validation and eventual use of these technologies in diagnosis and the incorporation of therapies in routine clinical practice.

## 1. Introduction

The human gastrointestinal tract is one of the most complex and diverse ecosystem known, with a plethora of fungi, viruses, bacteria and protists. This community of commensals usually dominated by bacteria is often referred to as microbiota, and their collective genome is termed the microbiome. The gut microbiota play critical role in the health of the host, which include but is not limited to the maturation of the immune system, the prevention of pathogenic infection, the alteration of intestinal morphology and angiogenesis, the fermentation of undigested polysaccharides and the synthesis and conversion of bioactive compounds [1,2,3,4].

Perturbations or dysbiosis of the gut microbiota as a result of diet, drug intake or environmental changes can result in severe health challenges with fatal outcomes. Diseases such as irritable bowel syndrome (IBS); obesity; inflammatory bowel disease (IBD), which includes ulcerative colitis (UC) and Crohn’s disease; cancer; and inflammatory disorders (diseases associated with abnormal function of the immune system and chronic inflammation) are associated with the perturbation of the gut microbiota [5,6]. An array of other diseases, such as neurodegenerative disorders (Alzheimer’s disease and the autism spectrum disorder), chronic kidney disease, diabetes and atherosclerotic cardiovascular disease, as summarized in Table 1, have been linked to the dysbiosis of the gut [7,8,9]. The focus of this review is centered on gastrointestinal diseases associated with the alteration of the gut microbiota, and recent technologies used in the study of gastrointestinal microbiota, with the view to identifying their potential applications in clinical practice.

## 2. Methods

The literature on gut microbiota, diseases associated with perturbation of the gut microbiota and technologies used in gut microbiome research were searched through Google Scholar and PubMed/MEDLINE. The final search date was 29 February, 2020. Search strings such as “gut microbiome”, “gastrointestinal microbiota”, “microbiota dysbiosis and metabolic syndrome” and “technologies in microbiota research” were used. The search comprised original and review articles written in English. Retrieved articles were reviewed and sorted to eliminate duplicates and unwanted articles.

## 3. Results and Discussion

**Figure 1:**
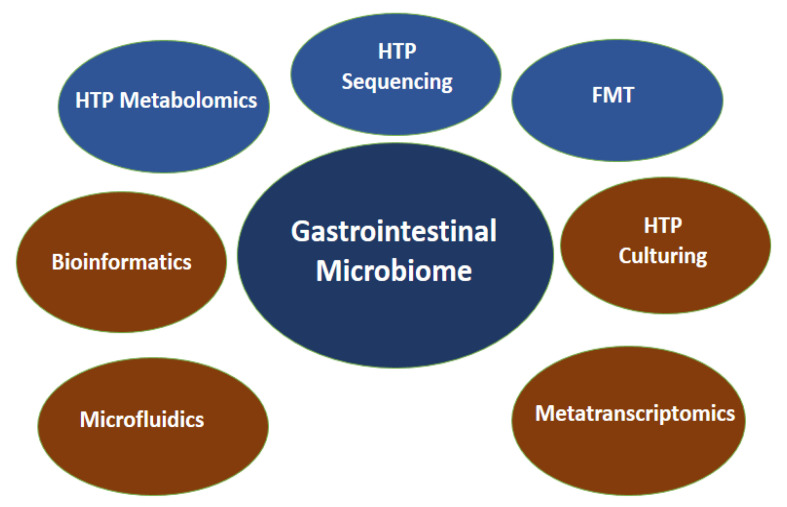
Various technologies used in the study of gastrointestinal microbiome. HTTP: high-throughput, FMT: fecal microbiota transplantation.

From the early start scientists used traditional culture and isolation techniques to study the flora of the body but today, improved methods including high-throughput culturing methods, high-throughput sequencing, microfluidics, human fecal transplant (Figure 1) approaches are being used in the study and treatment of the human microbiome ecosystem, so as to examine their role in inducing disease and to map out remedy against infective bacteria [16,17,18,19].

### 3.1. The Human Gut and Its Microbiota

Prior to its birth, it is presumed that the unborn is free of microbial flora, and that at birth, the infant first comes in contact with the resident microbial flora of the mothers’ vagina if birth was through the natural birth canal, or the microbial flora of the mothers’ skin if birth was through cesarean section [20,21,22]. Although some studies [23,24,25] have suggested the early inoculation of the fetus with bacteria and bacteria DNA through the placenta. The study by de Goffau et al. [26] reported that the human placenta has no microbiome. Detected bacteria were acquired during labor and delivery. After birth, according to the findings of Koenig et al. [27], there were apparent chaotic shifts of microbiome from that endowed with genes facilitating lactate utilization and plant polysaccharide metabolism mediated by milk-based diet to increase in *Bacteroidetes* initiated by introduction of solid food that prepares the infant gut for adult diet. However, in the findings of Differding et al. [28], the early introduction of infants to complementary food was associated with altered gut microbiota composition and butyric acid concentration, which have been previously identified as precursors to oxidative stress, immune disorder and obesity in childhood.

The microbiome of the adult gut accommodates various communities of phylotypes belonging to the phyla *Actinobacteria*, *Proteobacteria*, *Bacteroidetes*, *Fusobacteria*, *Firmicutes* and *Verrucomicrobia* [2]. Most of these phyla are present in the stomach, small intestine and colon. However, the colon is more populated with several genera belonging to the afore mentioned phyla, including the genus *Akkemansia* that belongs to the phylum *Verrucomicrobia*, which has been found to be limited in patients with obesity, inflammatory bowel disease and other metabolic syndromes, while it is in abundance in the biopsies of healthy individuals [2,29]. As has been reported in several studies, dietary types and pattern shapes and determines the diversity of the gut microbiome. In the submission of Amabebe et al. [30], high fat and carbohydrate diet builds a gut microbiota that is predominated by *Methanobrevibacter*, *Firmicutes* (*Clostridium*) and *Prevotella* and deficient in bacteria such as *Bacteroides*, *Lactobacillus*, *Akkermansia* and *Bifidobacterium*. Barone et al. [31], in their study brought to the fore the impact of modern Paleolithic diet (MPD) that consist of vegetables, seeds, lean meat, fruits, eggs, nuts and fish on the gut microbiome. They observed that the gut microbiome of urban Italians adhering to MPD showed an ample degree of biodiversity with high relative abundance of fat-loving and bile tolerant microorganisms. As have been mentioned earlier, perturbations or dysbiosis in combination with altered permeability are crucial mechanisms that mediate disease manifestation [32]. Fecal microbiota transplantation (FMT) has gained relevance in recent times in the treatment and correction of gut infections or disorders that might have resulted from the depletion of resident microbiota and infection by pathogenic bacteria. Huge successes have been recorded in FMT therapy, with about 92% efficacy reported in the treatment of recurrent *Clostridium difficile* infection [33]. In a recent study by Zou et al. [34], it was shown that patients with Crohn’s disease and ulcerative colitis that had FMT were in remission after three days of transplant with notable bacterial colonization of the gut. FMT therapy has been extended to the treatment of lifestyle and other diseases, such as diabetes, metabolic syndrome, Parkinson’s disease, obesity and cancer. FMT entails transfer of gut microbiota in feces of a healthy donor to recipient patient to correct/treat a disorder or gastrointestinal disease [35,36,37]. Although the level of success of this procedure, is yet to be wide spread due to some constraints identified by Cammarota et al. [38], including difficulties with donor recruitment, lack of dedicated centers and issues pertaining to safety monitoring and regulation, hence, the proposal for the provision of stool banks to bridge the gap of FMT in clinical practice.

The afore mentioned technique offers a natural option to routine medical treatments of chronic ailments by providing direct and effective remedy preventing dysbiosis in the host, thereby improving health conditions [39,40].

### 3.2. Technologies in Gastrointestinal Microbiome Study

Since the structure, composition and diversity of the human gut microbiota has been correlated with the health status of humans, it could be presumed that the future of combating certain ailments is through exploring individualized gastrointestinal microbiome as the gastrointestinal microbiome era heralds. In the past, scientists have used culture independent techniques such as electrophoresis based methods, including denaturing gradient gel electrophoresis (DGGE), temperature gradient gel electrophoresis (TGGE) and PCR based methods, such as terminal restriction fragment length polymorphism (T-RFLP) and random amplified polymorphic DNA (RAPD), to study the community structure, diversity and genetic relatedness of bacteria in communities. Fluorescence in situ hybridization (FISH) is a cytogenetic technique that has been used in the study of individual microbes within gut microbiota, such as *Listeria monocytogenes*, *Salmonella* species, *Helicobacter pylori* and *Yersinia enterocoliticai*, which are gut pathogens [41,42,43,44]. Russmann et al. [45] used FISH in the diagnosis of *Helicobacter pylori* cultured isolates, and the same technique was used to proffer antibiotic treatment options. These methods had a lot of drawbacks, including the need for specific probes, low resolution, specificity and sensitivity. However, advances in sequencing and culture technologies have paved the way to analyzing big data arising from exploration of the rich microbiome ecosystem of the gut, which is evident in several studies, as shown in Table 2. Such technologies are high-throughput sequencing, microfluidics, high-throughput metabolomics, assays engineered organoids derived from human stem cells and high-throughput culturing [46]. They have far reaching advantages over the older or traditional technology already mentioned, but with some limitations as well (summary in Table 3). The pros and cons of these technologies are described below.

### 3.3. 16S rRNA Gene Amplicon Sequencing

The in-depth study of the gut microbiome has been made possible through metagenomic approaches employing high-throughput sequencing technologies. Metagenomics entails the sequencing of total community DNA, which provides information on the richness, community structure and function of microbial species to be evaluated [54]. Sequencing of the hypervariable region of the 16S rRNA gene in combination with bioinformatics has been widely used to decipher the microbial composition of a community in an ecosystem like the gut. Using 16S rDNA illumina sequencing, Pires et al. [4] were able to characterize the gut microbiome of individuals living in the Amazon, which revealed huge variation in composition, compared to people living in industrialized settings. Similarly, Barone et al. [31] used information from 16S rRNA gene sequencing to explain gut microbiome response to a modern Paleolithic diet in a Western lifestyle context. Previous studies have also accessed and studied pediatric gut microbiome using 454 pyrosequencing of *16S rRNA* genes [27,55]. The use of 16S rRNA sequencing in evaluating the microbial composition of a microbiota has its various imperfections which whole genome shotgun sequencing (WGSS) has taken care of. WGSS has been used in several gastrointestinal microbiome studies. Vogtmann et al. [56] reported the reproducibility using WGSS in the study of the association of colorectal cancer and the human gut microbiome. Several bioinformatics platforms and tools, including Quantitative Insight Into Microbial Ecology (QIIME), Phylogenetic Investigation of Communities by Reconstruction of Unobserved States (PICRUSt), STatistical Analyses of Metagenomic Profiles (STAMP) [51], Linear Discriminant Analysis with Effect Size (LEfSe) [12] CLAssifier based on Reduced k-mers (CLARK), Mothur, Kraken [57] to mention a few that exist for analyzing the enormous genetic data that is generated from gastrointestinal microbiome studies. These tools help in predicting/assigning microbial taxonomy and give insight into the diversity, richness and composition of microbial species in a microbiota [58]. The enormous data obtained from metagenomic study of gut microbiota can be employed by clinicians for proper diagnosis or prediction of gastrointestinal diseases and guide antibiotic therapy in clinical settings. Vila et al. [6], demonstrated through analyzing metagenomic data of the gut microbiota of patients that IBD could be differentiated from IBS with microbial taxonomic makers, since both conditions have overlapping clinical manifestation that requires colonoscopy (an invasive procedure) for an accurate diagnosis by a clinician. Furthermore, in the same study, they were able to capture, from the same data, the resistome of the patients. A major limitation of this approach is the bias in the composition of databases to which comparisons are made.

### 3.4. Microfluidics

**Figure 2:**
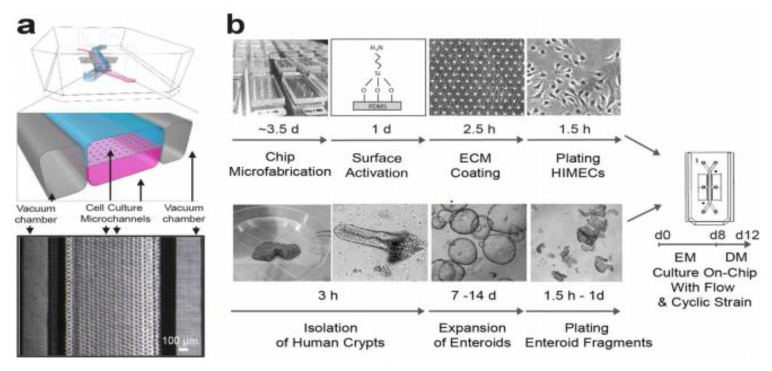
Primary human intestine chip manufacturing outline. (**a**) A cross-sectional view from the top of the chip and a phase contrast micrograph of the chip viewed from the bottom showing the upper (epithelial; blue) and lower (microvascular; pink) cell culture microchannels separated by a porous, Extracellular Matrix (ECM)-coated, Polydimethylsiloxane (PDMS) membrane sandwiched in-between. (**b**) An outline in developing microfuidic co-cultures of primary human intestinal epithelium and intestinal microvascular endothelium in the intestine chip. Source: Kasendra et al. [60] (*Scientific Reports*, Springer Nature) Licensed under CC BY 4.0.

A major challenge in gastrointestinal microbiome research is the existence of the microbiota as a community in which most members are yet to be cultivated thus making it difficult to identify what species is doing what within the ecosystem. Microfluidics technology is providing a platform where single microbial cells within the gastrointestinal microbiota can be tracked, studied and manipulated. Liu and Walther-Antonio [58] identified two powerful microfluidics that have the potential applications in cell sorting, cell culture, cell screening, genome application, metabolic screening/analyses and gene expression. A recent study by Chijiwa et al. [52] used single cell sequencing based on an SAG-gel platform that employed microfluidic droplet generator to unravel the metabolic function of uncultivated bacterial species in intestinal microbiome, in which the fermentation of dietary fiber resulting in the production of short-chain fatty acid from ingested fibers was evaluated. This technique enabled the thorough study of specific bacterial species and deciphering their specific function that contributes to the health of the gastrointestinal tract. Another interesting innovation in the area of microfluidics in microbiome research is the development of organs on chip. This has allowed the design of experiments that captures minute complexities of microenvironment with extraordinary resolution, giving details of the microbial diversity, structure and functions of the microbiota of specific organs of the human system [59]. The development of a primary human small intestine on a chip using biopsy derived organoids by Kasendra et al. [60] (as shown in Figure 2), in which they suggested that its potential use in the study of infection, metabolism, drug pharmacokinetics and nutrition can be a viable and efficient tool in studying human gastrointestinal microbiota. Another principal application of microfluidics is in the study of antibiotic susceptibility of bacterial cells in real time. Cama et al. [61] in their study demonstrated the rapid quantification of antibiotic accumulation in Gram negative bacteria.

By employing a combination of time-lapse auto-fluorescence microscopy and single-cell microfluidics, this technology could help reduce the overprescription of antibiotics, which is known to drive resistance. Beyond the aforementioned potential application of microfluidics, it also has clinical application in understanding the pathogenesis of gastrointestinal diseases, diagnosis, drug delivery and personalized or individualized medicine.

### 3.5. High-Throughput Metabolomics

High-throughput metabolomics (HTM) is becoming a popular method for studying various metabolites resulting from activities of bacterial populations in microbiota. Metabolomics is the process of assessing the metabolite profile in any given sample or ecosystem [62]. This has been made possible by using methods such as high throughput mass spectrometry. Koening et al. [27] used gas chromatography-mass spectrometry (GC-MS) to measure the concentration of short chain fatty acids (acetate, butyrate and propionate) in fecal samples, which was then correlated with bacterial diversity of the gut microbiota of infants. Similarly, Pires et al. [4] used mass spectrometry with direct infusion (DI-MS) on a Fourier transform ion cyclotron resonance instrument, to evaluate the chemical ecology of the gut environment of urban and rural dwellers of the Amazon. The use of HTM in gastrointestinal microbiome study has been used to demystify the role of short chain fatty acid (propionic acid) in ameliorating multiple sclerosis disease in humans. Duscha et al. [63] investigated variations in microbiota and their associated short chain fatty acid metabolites and the effect of dietary propionic acid on immune-regulatory elements using high-performance liquid chromatography-tandem mass spectrometry (LC-MS/MS). It was revealed that propionic acid was greatly reduced in the serum and feces of multiple sclerosis patients compared with healthy controls. Conversely, the accumulation of specific short chain fatty acids has been associated with obesity, because they become additional source of energy, thereby altering the balance of energy regulation [64]. Other metabolites, including polyunsaturated fatty acids linked with regulating several processes within the brain, bile acids, such as lithocholic acid, ursodeoxycholic acid and tauroursodeoxycholic acid and amino acid neurotransmitters such as glycine, aspartic acid, glutamic acid and gamma-aminobutyric acid (GABA), are metabolic products of the activities of gut microbiota, which have been profiled by mass spectrometry based metabolomics [65]. Wilson and Forse [66] also developed an electronic-nose technology for early disease detection in microbial dysbiosis. These electronic-nose technologies have multi-sensor arrays and are able to analyze chemicals. Their invention could detect new groups of volatile organic compounds that are biomarkers metabolites also known as dysbiosis-associated disease markers, thereby providing a link between human ailments and resident microbes. The technology is noninvasive as it uses breath as sampling method. Gut metabolome is undoubtedly a peculiar candidate for the clinical diagnosis and management of gastrointestinal diseases. However, it comes with attendant limitations. Smirnov et al. [67] identified some limitations of metabolomics in gut microbiota research, including problems with sample handling, resulting in the loss of some metabolites due to freezing and thawing; drawbacks in personalized medicine/nutrition, due to the existence of variability in human microbiota and their metabolites; choice of adequate animal model and equipment. Improvement in sample handling and processing will prevent the loss of vital metabolites that otherwise would have not been accounted for. Furthermore, creating a database of metabolites associated with various members of the gut microbiota will enhance the use of gut metabolome in assigning biomarkers for the purpose of diagnosis, treatment and management of gastrointestinal diseases by physicians.

### 3.6. Metatranscriptomics

Metatranscriptomics is another technique that has been employed in gut microbiota studies leveraging the technological advances in RNA sequencing (RNA-seq) [68]. Metatranscriptomics entails retrieving, sequencing and analyzing total messenger RNA (mRNA) or microRNA (from a microbial ecosystem), to ascertain what genes are expressed within that community [69]. In practice, the retrieved or extracted RNA is converted into a complimentary DNA (cDNA) using a reverse transcriptase and oligo (dT) primers or random hexamers, after which libraries are constructed and sequenced. However, semi direct RNA sequencing, bypassing the conversion of RNA to cDNA, has also been developed. Metatranscriptomics is apt in human gut microbiota exploration, as it shows the real-time functional activities of microbiomes and is better positioned in associating gut microorganisms with host performance [70]. Furthermore, it provides a window through which active pathways are identified, and shows how expressed functions have a role in disease severity and progression [71]. This technology also gives insight into the interaction of the gut microbiota and mucosal immune system, which can help physicians track malfunctions in the host’s physiology [68].

### 3.7. High-Throughput Culturing

**Figure 3:**
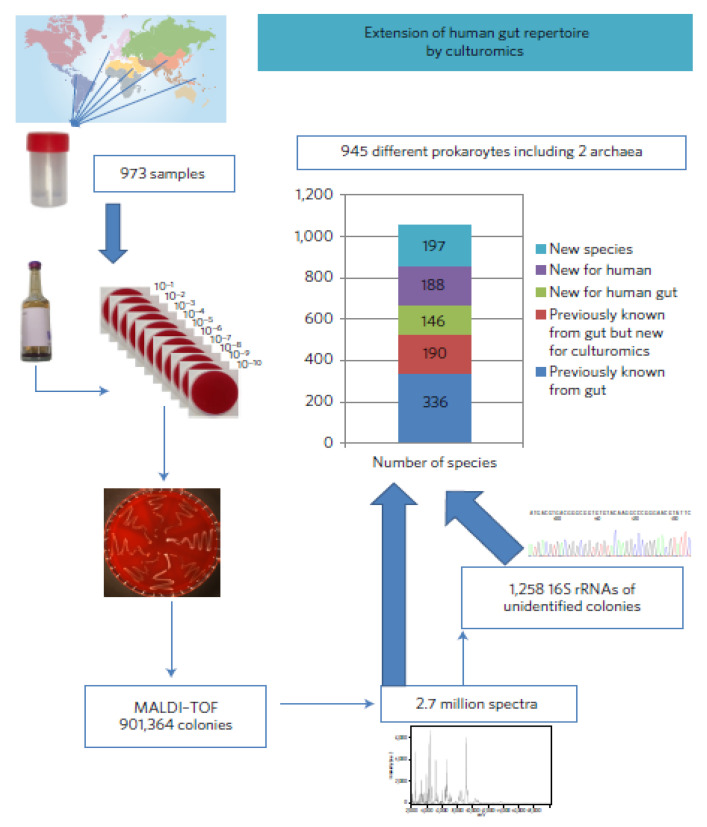
A stepwise outline of culturomics, enabling the culture of previously uncultured bacterial species in the human gut. Source: Lagier et al. [72] (*Nature Microbiology*, Macmillan Publishers Limited) Licensed under CC BY 4.0.

Several bacterial culture techniques have been developed overtime making it possible to culture a reasonable number of gut bacteria that had not been cultivated in the past. One such culture technique is culturomics. Culturomics, according to Lagier et al. [72], is a culturing technique that employs multiple culture conditions and matrix-assisted laser desorption/ionization time of flight (MALDI-TOF) and 16S rRNA gene amplification/sequencing for identification, as shown in Figure 3. Traore et al. [73] isolated 1162 bacteria strains by culturomics in a study that compared the gastrointestinal microbiota of Africans to the West. Goodman et al. [74] reported the use of gut microbiota medium (GMM) with high-throughput anaerobic culturing techniques in combination with metagenomics in characterizing extensive personal human gut microbiota culture collection and manipulation in gnotobiotic mice. Similarly, Lau et al. [49] revealed that using culture-enriched molecular profiling consisting of 66 culture conditions in conjunction with 16S rRNA gene sequencing was able to yield a robust data on the diversity of the microbiota that were present in sampled human feces. High-throughput culture methods have obvious advantages of enhancing the culturability of otherwise ‘non-culturable’ bacterial population, hence, giving room for the in-depth study of identified species. This technique is quite elaborate requiring specialized laboratories and it is space and time consuming. Harnessing the enormous potentials of high-throughput culturing techniques will provide clinicians with the platform for precise treatment of gut associated diseases resulting from perturbation, since implicated microbiota members can be cultured. Additionally, in the formulation and administration of probiotics, this technique can be useful. Furthermore, the morphology, physiology and biochemistry of individual microorganism can be studied and their response/interaction with drugs easily evaluated, thereby enabling the proper treatment of gut diseases.

## 4. Future Perspectives and Conclusions

Although research in gut microbiota is still evolving, already available data and methods of exploring this complex ecosystem has helped to sharpen our knowledge and understanding of the microbiome and how it affects human health. Several studies and technologies that have focused on interactions between the gut microbiome and diet have revealed how the introduction of new micro-organisms and their change over time have opened up opportunities for their future intervention, as well as diagnostic tools based on the microbiome. These methods have been used to identify the yet-to-be cultured microbes of the gut microbiota, using the activity of various microbial metabolites for the purpose of pathogen identification. In addition, gut microbiome studies have been used in fecal microbial transplant for the treatment and correction of gut infections/disorders.

These techniques have therefore been proven to provide efficient information on the gut microbiome and how it evolves over time, thereby generating rich data sets that have been useful for treatment of yet to be identified pathogens, while enabling faster and more accurate diagnosis even from non-invasive sampling. Finally, the gut microbiome research has upped the ante on genomic technologies, because genomes of the gut microbiomes are sequenced to give us a better understanding of the gut microbiome.

## Figures and Tables

**Table 1:** Summary of Diseases/Metabolic Syndrome, Microbial Indicators of Healthy and Dysfunctional Gut.

Subject	Metabolic Syndrome/Disease	Correlated Clinical Indicator	Indicator Microbe (Healthy Metabolic State)	Indicator Microbe (Dysfunctional Metabolic State)	Reference
Adult (Male & Female)	Prediabetes or type 2 diabetes mellitus	Impaired lipid & glucose metabolism	*Clostridia* and *Rikenellaceae* members	*Holdernania* & *Blautia* genera	[10]
Adult	Obesity	NI	Balance population of *Bacteroidetes* & *Firmicutes*	Few Bacteroidetes & more Firmicutes	[11]
Adult	Alzheimer’s Disease	Low Mini-Mental State Examination score, APOE ε4 carriers, high Clinical Dementia Rating and Activity of Daily Living scores	Normal gut microbiota population comprising *Firmicutes, Proteobacteria, Bacteroidetes* and *Actinobacteria*	Significant decrease in the population of *Negativicutes* and *Bacteroidia*	[12,13]
Children	Autism	NI	Moderate level of *Clostridium histolyticum,* normal population of *Firmicutes, Bacteroidetes*	Lower levels of *Prevotella, Coprococcus, Veillonellaceae, Firmicutes and Bifidobacterium and* higher levels of *Clostridium histolyticum, Desulfovibrio, Lactobacillus, Sarcina Clostridium, Bacteroidetes and Caloramator*	[14]
Adult	Early chronic kidney disease	abnormal kidney structure, urinary albumin excretion rate ≥30 mg/24 h, glomerular filtration rate, 30–90 mL/minute/1.73 m^2^	Abundance of *Roseburia* and other genera	Abundance of *Ruminococcus* and other genera	[9]
Adult	Atherosclerotic cardiovascular disease	stable angina, unstable angina, or acute myocardial infarction, ≥50% stenosis in single or multiple vessel	Higher population of *Bacteroides* and *Prevotella*	Relative reduction in *Bacteroides* and *Prevotella* and enrichment in *Streptococcus* spp. and *Escherichia*, *Klebsiella* spp., *Enterobacter aerogenes*	[15]

NI: Not Indicated.

**Table 2:** Studies on Microbiome, Outcomes and Methods Employed.

Subject	Methods Employed	Outcome	Reference
Association between breast milk oligosaccharides and fecal microbiota in healthy breast fed infants	16S rRNA genes sequencing of V4 region using the Illumina Hiseq 2000 platform, porous graphitized carbon-ultra high-performance liquid chromatography (PGC-UPLC-MS) and bioinformatics (QIIME)	Microbiota composition strongly influenced by infant age, associated mode of delivery and breast milk	[47]
Dynamics and stabilization of the human gut microbiome during the first year of life	Metagenomics (DNA extraction from stool samples and preparation of DNA library using Illumina Hiseq2000) and bioinformatics (SOAPdenovo2, GeneMark v2.7, NCBI database)	Nutrition has a far reaching influence on infant microbiota composition and function with halting of breast-feeding other than introduction of solid food	[48]
Determining the diversity of human gut microbiota	Culture with enrichment, 16S rRNA gene sequencing of V3 region using the Illumina Miseq platform and bioinformatics (QIIME)	Use of enriched culture method enhanced the culturability of bacteria identified by 16S sequencing of the microbiota of the human gut	[49]
Impact of diet during pregnancy on maternal microbiota clusters and its influence on neonatal microbiota and infant growth during the first 18 months of life	16S rRNA gene sequencing of V3-V4 region using Miseq Illumina platform. Bioinformatics (QIIME, LEfSe, Calypso online platform)	Diet is an important perinatal factor in the initial phase of life and have significant impact on neonatal microbiome	[50]
Heritable components of the human fecal microbiome are associated with visceral fat	Measuring of body composition by dual-energy X-ray absorptiometry, 16S rRNA gene sequencing of V4 region on Illumina Miseq platform and bioinformatics (QIIME 1.7.0, PICRUSt v1.0.0, STAMP)	There was significant association of adiposity-OTU abundance with host genetic variations indicating possible role of host genes in influencing the link between obesity and fecal microbiome	[51]
Succession of microbial consortia in the developing infant gut microbiome	454-pyrosequencing of 16S rRNA gene, GC-MS analysis of SCFA, quantitative PCR and bioinformatics (QIIME, MG-RAST, NCBI database)	Revealed shifts in microbiome associated with life events	[26]
Identification of uncultured bacteria that are metabolic responders in a microbiota	Massively parallel single-cell genome sequencing technique (SAG-gel Platform), 16S rRNA gene sequencing of V3-V4 using Illumina Miseq 2 x 300bp platform and bioinformatics (QIIME2 v.2019.1). Determination of the concentration of SCFA was done by GC-mass spectrophotometry	Functions of uncultured bacteria in the microbiota were elucidated	[52]
Study of human gut colonization linked to in utero by microbial communities in the amniotic fluid and placenta	Culture, Gradient Gel Electrophoresis (DGGE), 16S rRNA gene pyrosequencing of V1-V3 region, quantitative PCR and bioinformatics (PICRUSt, QIIME, LEfSe)	The microbiota composition of infant gut at the age of 3-4 days begins to look like that detected in colostrum hence, the presumption that colonization is initiated prenatally by a distinct microbiota in the amniotic fluid and placenta	[53]

**Table 3:** Summary of the Potential Clinical Application of Various Technologies and Their Advantages and Disadvantages.

Technology/Methodology	Advantage	Disadvantage	Potential Clinical Application
Metagenomics (High-through sequencing)	Provides information on culturable and ‘non-culturable’ or yet to be cultured microorganisms. Captures both viable and unviable species of microorganisms. Essential details of diversity and community structure of the gut microbiota is provided	Further studies on microorganisms present in the microbiota is not possible since direct extraction of DNA is employed restricting physical access to the microorganisms.	Could be used by clinicians for the proper diagnosis of gastrointestinal diseases with overlapping clinical presentation. Or for identifying microbiological markers that predict the presence of certain diseases.
High-throughput Metabolomics	Provides information on the various metabolites of gut resident microorganisms and how it correlates to disease conditions. Specific metabolites identified could serve as biomarkers Can be used for measuring and evaluating the effect of dietary intake on the gut microbiota	Loss of metabolites of some members of the microbiota due to sample handling. Drawback in its use for personalized medicine/nutrition because of the existence of variability in human microbiota and their metabolites.	Monitoring metabolites of gut microbiota using high-throughput metabolomics can help in the early diagnosis and management of metabolic syndromes that has been linked with the gut microbiota. Can guide physicians on recommending dietary intake to patients.
High-throughput Metatranscriptomics	Captures active members of the microbiota Gives insight into the functions of various members of the gut microbiota Can provide information on how members of the microbiota respond to changes within their environment	Since RNA is not as stable as DNA, handling of sample can results in biases in finial results analyzed. There is still a shortfall in metadata in repositories to which the enormous data generated from metatranscriptomics of the gut can match since this technology is still evolving	Can identify how the function of a microbe in the gut influence the severity or progression of a disease Can be used to monitor the interaction of the gut microbiota and host’s mucosal immune system
Microfluidics	Provide miniaturized platform for in vitro simulation, cultivation and manipulation of gut microbiota. Make possible selective targeting and culture of important members of the gut microbiota. Permit the combination of culture, DNA extraction, amplification and sequencing on a single platform.	Human gut on chip might not give optimal performance as in natural human gut.	This technology can be deployed clinically to monitor perturbation of gut microbiota in good time and enable precision in intervention by manipulating and stimulating the growth of beneficial or essential gut health promoting bacteria. Microfluidics in microbiome studies can guide in the prescription of antibiotics.
High-throughput Culturing	Culture gives access to the in-depth study of individual microorganisms that are cultured from the gut microbiota providing information on structure, morphology, physiology, growth conditions, inter & intra species interactions. Culture captures only viable bacteria population. Enable enumeration of bacteria species present	Laborious and time consuming. Limited number of members of the microbiota are accounted for since majority of them are ‘non-culturable’ or yet to be cultured. Technique may be expensive due to the array of materials and specialized laboratory needed.	Could provide avenue for precise treatment of gut diseases resulting from dysbiosis of specific species of bacteria and enable formulation of probiotics

## References

[1] Matsuki T., Tanaka R. (2014). Function of the human gut microbiota. The Human Microbiota and Microbiome.

[2] Blaut M. (2018). Composition and function of the gut microbiome. The Gut Microbiome in Health and Disease.

[3] Valdes A., Walter J., Segal E., Spector T. (2018). Role of the gut microbiota in nutrition and health. BMJ.

[4] Pires E.S., Hardoim C.C.P., Miranda K.R., Secco D.A., Lobo L.A., de Carvalho D.P., Han J., Borchers C.H., Ferreira R.B.R., Salles J.F. (2019). The gut microbiome and metabolome of two riparian communities in the amazon. Front. Microbiol..

[5] Karkman A., Lehtimäki J., Ruokolainen L. (2017). The ecology of human microbiota: Dynamics and diversity in health and disease. Ann. N. Y. Acad. Sci..

[6] Vila A.V., Imhann F., Collij V., Jankipersadsing S.A., Gurry T., Mujagic Z., Kurilshikov A., Bonder M.J., Jiang X., Tigchelaar E.F. (2018). Gut microbiota composition and functional changes in inflammatory bowel disease and irritable bowel syndrome. Sci. Transl. Med..

[7] Wang X., Sun G., Feng T., Zhang J., Huang X., Wang T., Xie Z., Chu X., Yang J., Wang H. (2019). Sodium oligomannate therapeutically remodels gut microbiota and suppresses gut bacterial amino acids-shaped neuroinflammation to inhibit Alzheimer’s disease progression. Cell Res..

[8] Kang D.-W., Adams J.B., Gregory A.C., Borody T., Chittick L., Fasano A., Khoruts A., Geis E., Maldonado J., McDonough-Means S. (2017). Microbiota Transfer Therapy alters gut ecosystem and improves gastrointestinal and autism symptoms: An open-label study. Microbiome.

[9] Hu Q., Wu K., Pan W., Zeng Y., Hu K., Chen D., Huang X., Zhang Q. (2020). Intestinal flora alterations in patients with early chronic kidney disease: A case-control study among the Han population in southwestern China. J. Int. Med. Res..

[10] Lippert K., Kedenko L., Antonielli L., Gemeier C., Leitner M., Kautzky-Willer A., Paulweber B., Hackl E., Kedenko I. (2017). Gut microbiota dysbiosis associated with glucose metabolism disorders and the metabolic syndrome in older adults. Benef. Microbes.

[11] Ley R.E., Turnbaugh P.J., Klein S., Gordon J.I. (2006). Microbial ecology: Human gut microbes associated with obesity. Nature.

[12] Zhuang Z.-Q., Shen L.-L., Li W.-W., Fu X., Zeng F., Gui L., Lv Y., Cai M., Zhu C., Tan Y.-L. (2018). Gut Microbiome is Altered in Patients with Alzheimer’s Disease. J. Alzheimer’s Dis..

[13] He Y., Li B., Sun D., Chen S. (2020). Gut microbiota: Implications in Alzheimer’s disease. J. Clin. Med..

[14] Li Q., Han Y., Dy A.B.C., Hagerman R.J. (2017). The Gut Microbiota and Autism Spectrum Disorders. Front. Cell. Neurosci..

[15] Jie Z., Xia H., Zhong S.-L., Feng Q., Li S., Liang S., Zhong H., Liu Z., Gao Y., Zhao H. (2017). The gut microbiome in atherosclerotic cardiovascular disease. Nat. Commun..

[16] Feeney A., Sleator R.D. (2012). The human gut microbiome: The ghost in the machine. Future Microbiol..

[17] Khosravi A., Mazmanian S.K. (2013). Disruption of the gut microbiome as a risk factor for microbial infections. Curr. Opin. Microbiol..

[18] Sommer M.O. (2015). Advancing gut microbiome research using cultivation. Curr. Opin. Microbiol..

[19] Dabke K., Hendrick G., Devkota S. (2019). The gut microbiome and metabolic syndrome. J. Clin. Investig..

[20] Barko P.C., McMichael M.A., Swanson K.S., Williams D.A. (2018). The gastrointestinal microbiome. J. Vet. Intern. Med..

[21] Dominguez-Bello M.G., Costello E.K., Contreras M., Magris M., Hidalgo G., Fierer N., Knight R. (2010). Delivery mode shapes the acquisition and structure of the initial microbiota across multiple body habitats in newborns. Proc. Natl. Acad. Sci. USA.

[22] Shao Y., Forster S.C., Tsaliki E., Vervier K., Strang A., Simpson N., Kumar N., Stares M.D., Rodger A., Brocklehurst P. (2019). Stunted Microbiota and Opportunustic pathogens colonization in caesarean-section birth. Nature.

[23] Satokari R., Gronroo T., Laitinen K., Salminen S., Isolauri E. (2009). Bifidobacterium and Lactobacillus DNA in the human placenta. Lett. Appl. Microbiol..

[24] Aagaard K., Ma J., Antony K.M., Ganu R., Petrosino J., Versalovic J. (2014). The placenta harbors a unique microbiome. Sci. Transl. Med..

[25] Yu K., Rodriguez M.D., Paul Z., Gordon E., Rice K., Triplett W.E., Keller-Wood M., Wood C.E. (2019). Proof of principle: Physiological transfer of small numbers of bacteria from mother to fetus in late-gestation pregnant sheep. PLoS ONE.

[26] De Goffau M.C., Lager S., Sovio U., Gaccioli F., Cook E., Peacock S.J., Parkhill J., Charnock-Jones D.S., Smith G.C.S. (2019). Human placenta has no microbiome but can contain potential pathogens. Nature.

[27] Koenig J.E., Spor A., Scalfone N., Fricker A.D., Stombaugh J., Knight R., Angenent L.T., Ley R.E. (2011). Succession of microbial consortia in the developing infant gut microbiome. Proc. Natl. Acad. Sci. USA.

[28] Differding M.K., Benjamin-Neelon S.E., Hoyo C., Østbye T., Mueller N.T. (2020). Timing of complementary feeding is associated with gut microbiota diversity and composition and short chain fatty acid concentrations over the first year of life. BMC Microbiol..

[29] Cozzolino A., Vergalito F., Tremonte P., Iorizzo M., Lombardi S.J., Sorrentino E., Luongo D., Coppola R., Marco R.D., Succi M. (2020). Preliminary evaluation of the safety and probiotic potential of *Akkermansiamuciniphila* OSM 22959 in comparison with *Lactobacillus rhamnosus* GG. Microorganisms.

[30] Amabebe E., Robert F.O., Agbalalah T., Orubu E.S.F. (2020). Microbial dysbiosis-induced obesity: Role of gut microbiota in homeostasis of energy metabolism. Br. J. Nutr..

[31] Barone M., Turroni S., Rampelli S., Soverini M., D’Amico F., Biagi E., Brigidi P., Troiani E., Candela M. (2019). Gut microbiome response to a modern Paleolithic diet in a western lifestyle context. PLoS ONE.

[32] Caviglia G.P., Rosso C., Ribaldone D.G., Dughera F., Fagoonee S., Astegiano M., Pellicano R. (2019). Physiopathology of intestinal barrier and the role of Zonulin. Minerva Biotecnol..

[33] Kim K.O., Gluck M. (2019). Fecal Microbiota Transplantation: An Update on Clinical Practice. Clin. Endosc..

[34] Zou M., Jie Z., Cui B., Wang H., Feng Q., Zou Y., Zhang X., Yang H., Wang J., Zhang F. (2019). Faecalmicrobiota transplantation results in bacterial strain displacement in patients with inflammatory bowel diseases. FEBS Open Bio.

[35] Bibbò S., Settanni C.R., Porcari S., Bocchino E., Ianiro G., Cammarota G., Gasbarrin A. (2020). Fecal Microbiota Transplantation: Screening and Selection to Choose the Optimal Donor. J. Clin. Med..

[36] Sbahi H., Di Palma J.A. (2016). Faecalmicrobiota transplantation: Applications and limitations in treating gastrointestinal disorders. BMJ Open Gastroenterol..

[37] Antushevich H. (2019). Faecalmicrobita transplantation in disease therapy. Clin. Chim. Acta.

[38] Cammarota G., Laniro G., Kelly C.R., Mullish B.H., Allegretti J.R., Kassam Z., Putignani L., Fischer M., Keller J.J., Castello S.P. (2019). International consensus conference on stool banking for faecalmicrobiota transplantation in clinical practice. Gut.

[39] Belizário J.E., Faintuch J., Garay-Malpartida M. (2018). Gut Microbiome Dysbiosis and Immunometabolism: New Frontiers for Treatment of Metabolic Diseases. Mediat. Inflamm..

[40] Wong A.C., Levy M. (2019). New approaches to microbiome-based therapies. mSystems.

[41] Guimaraes N., Azevedo N.F., Figueiredo C., Keevil C.W., Vieira M. (2007). Development and application of a novel peptide nucleic acid probe for the specific detection of Helicobacter pylori in gastric biopsy specimen. Clin. Microbiol..

[42] Baysal A.H. (2014). Comparison of conventional culture method and fluorescent in situ hybridization technique from detection of Listeria Spp. In ground beef, turkey and chicken breast fillets in Izmir, Turkey. J. Food Prot..

[43] Becattini S., Littmann E.R., Carter R.A., Kim S.G., Morjaria S.M., Ling L., Gyaltshen Y., Fontana E., Taur Y., Leiner I.M. (2017). Commensal Microbesprovide First Line Defence Agaimst Listeria monocytogenes Infection. J. Exp. Med..

[44] Prudent E., Raoult D. (2019). Fluorescent in situ hybridization, a complementary molecular tool for the clinical diagnosis of infectious diseases by intracellular and fastidious bacteria. FEMS Microbiol. Rev..

[45] Russmann H., Adler K., Haas R., Gebert B., Koletzko S., Heesemann J. (2001). Rapid and accurate Determination of genotypic clarithromycin resistance in cultured Helicobacter pylori by fluorescent in situ hybridization. J. Clin. Microbial..

[46] Arnold J.W., Roach J., Azcarate-Peril M.A. (2016). Emerging technologies for gut microbiome research. Trends Microbiol..

[47] Borewicz K., Gu F., Saccenti E., Hechler C., Beijers R., de Weerth C., van Leeuwen S.S., Schols H.A., Smidt H. (2020). The association between breast milk oligosaccharides and faecal microbiota in healthy breastfed infants at two, six, and twelve weeks of age. Sci. Rep..

[48] Backhed F., Roswall J., Peng Y., Feng Q., Jia H., Kovatcheva P.-D., Li Y., Xia Y., Xie H., Zhong H. (2015). Dynamics and Stabilization of the Human Gut Microbiome during the First Year of Life. Cell Host Microbe.

[49] Lau J.T., Whelan F.J., Herath I., Lee C.H., Collins S.M., Bercik P., Surette M.G. (2016). Capturing the diversity of the human gut microbiota through culture-enriched molecular profiling. Genome Med..

[50] García-Mantrana I., Selma-Royo M., González S., Parra-Llorcac A., Martínez-Costad C., Collado M.C. (2020). Distinct maternal microbiota clusters are associated with diet during pregnancy: Impact on neonatal microbiota and infant growth during the first 18 months of life. Gut Microbes.

[51] Beaumont M., Goodrich J.K., Jackson M.A., Yet I., Davenport E.R., Vieira S.-S., Debelius J., Pallister T., Mangino M., Raes J. (2016). Heritable components of the human fecal microbiome are associated with visceral fat. Genome Biol..

[52] Chijiwa R., Hoskawa M., Kogawa M., Nishikawa Y., Ide K., Sakanashi C., Takahashi K., Takeyama H. (2020). Single-cell genomics of uncultured bacteria reveals dietary fiber responders in the mouse gut microbiota. Microbiome.

[53] Collado M.C., Rautava S., Aakko J., Isolauri E., Salminen S. (2016). Human gut colonization may be initiated in utero by distinct microbial communities in the placenta and amniotic fluid. Sci. Rep..

[54] Albenberg L., Kelsen J. (2016). Advances in gut microbiome research and relevance to pediatric diseases. J. Pediatr..

[55] Saulnier D.M., Riehle K., Mistretta T.A., Diaz M.-A., Mandal D., Raza S., Weidler E.M., Qin X., Coarfa C., Milosavljevic A. (2011). Gastrointestinal microbiome signatures of pediatric patients with irritable bowel syndrome. Gastroenterology.

[56] Vogtmann E., Hua X., Zeller G., Sunagawa S., Voigt A.Y., Hercog R., Weidler E.M., Qin X., Coarfa C., Milosavljevic A. (2016). Colorectal cancer and the human gut microbiome: Reproducibility with whole genome shotgun sequencing. PLoS ONE.

[57] Siegwald L., Caboche S., Even G., Viscoglisoi E., Audebert C., Chabe M. (2019). The impact of bioinformatics pipelines on microbiota studies: Does the analytical “Microscope” affect the biological interpretation?. Microorganisms.

[58] Liu Y., Walther-Antonio M. (2017). Microfluidics: A new tool for microbial single cell analyses in human microbiome studies. Biomicrofluidics.

[59] Udayasuryan B., Slade D.J., Verbridge S.S. (2019). Microfluidics in Microbiome and Cancer Research.

[60] Kasendra M., Tovaglieri A., Sontheimer A.-P., Jalili-Firoozinezhad S., Bein A., Chalkiadaki A., Scholl W., Zhang C., Rickner H., Bein A. (2018). Development of a primary human Small Intestine-on-a-Chip using biopsy-derived organoids. Sci. Rep..

[61] Cama J., Voliotis M., Metz J., Smith A., Iannucci J., Keyser U.F., Atanasova K.T., Pagliara S. (2020). Single-cell microfluidics facilitates the rapid quantification of antibiotic accumulation in Gramnegative bacteria. Lab Chip.

[62] Sarangi A.N., Goel A., Aggarwal R. (2019). Methods for studying gut microbita: A primer for physicians. J. Clin. Exp. Hepatol..

[63] Duscha A., Gisevius B., Hirschberg S., Yissachar N., Stangl G.J., Eilers E., Bader V., Haase S., Kaisler J., David C. (2020). Propionic acid shapes the multiple sclerosis disease course by an immunomodulatory mechanism. Cell.

[64] Murugesan S., Nirmalkar K., Hoyo-Vadillo C., García-Espitia M., Ramírez-Sánchez D., García-Mena J. (2017). Gut microbiome production of short-chain fatty acids and obesity in children. Eur. J. Clin. Microbiol. Infect. Dis..

[65] Luan H., Wang X., Cai Z. (2017). Mass spectrometry-based metabolomics: Targeting the crosstalk between gut microbiota and brain in neurodegenerative disorders. Mass Spectrom. Rev..

[66] Wilson A.D., Forse L.B. (2019). Development of Electronic-Nose technologies for early disease detection based on microbial dysbiosis. Proceedings.

[67] Maier T.V., Walker A., Heinzmann S.S., Forcisi S., Martinez I., Walter J., Kopplin P.S. (2016). Challenges of metabolomics in human gut microbiota research. Int. J. Med. Microbiol..

[68] Bashiardes S., Zilberman-Schapira G., Elinav E. (2016). Use of Metatranscriptomics in Microbiome Research. Bioinform. Biol. Insights.

[69] Gosalbes M.J., Durban A., Pignatelli M., Abellan J.J., Jimenez-Hernandez N., Perez-Cobas A.E., Latorre A., Moya A. (2011). Metatranscriptomic Approach to Analyze the Functional Human Gut Microbiota. PLoS ONE.

[70] Li F., Hitch T.C.A., Chen Y., Creevey C.J., Guan L.L. (2019). Comparative metagenomic and metatranscriptomic analyses reveal the breed effect on the rumen microbiome and its associations with feed efficiency in beef cattle. Microbiome.

[71] Shakya M., Lo C.C., Chain P.S.G. (2019). Advances and Challenges in Metatranscriptomic Analysis. Front. Genet..

[72] Lagier J.-C., Khelaifia S., Alou M.T., Ndongo S., Dione N., Hugon P., Caputo A., Cadoret F., Traore S.I., Secck E.H. (2016). Culture of previously uncultured members of the human gut microbiota by culturomics. Nat. Microbiol..

[73] Traore S.I., Bilen M., Cadoret F., Khelaifa S., Million M., Raoult D., Lagier J.C. (2019). Study of huma gastrointestinal microbiota by culturomics in Africa. Med. Sante Trop..

[74] Goodman A.L., Kallstrom G., Faith J.J., Reyes A., Moore A., Dantas G., Gordon J.I. (2011). Extensive personal human gut microbiota culture collection characterized and manipulated in gnobiotic mice. Proc. Natl. Acad. Sci. USA.

